# A Text-Book of Legal Medicine and Toxicology

**Published:** 1903-09

**Authors:** 


**A Text-book of Legal Medicine and Toxicology.** Edited by FREDERICK PETERSON, M.D., and WALTER S. HAINES, M.D. Vol. I. Pp. 730. London: W. B. Saunders & Co. 1903.

Several works on this subject have been issued during the last few years from the English and American Press. Some of these works have been highly condensed, in supposed aid of the student for examination purposes; whilst, perhaps, others have treated some points at fuller length than usual. One or two of these works have been issued with very excellent and highly artistic illustrations, which photography and modern colour processes have enabled the authors to add to their books in a liberal way which had been previously impossible owing to the costliness of production. The present work also has some plates of this kind, but they are rather harsh in the tone of the colours used.

Vol. I. only has reached us at present. It consists of a number of separate articles written by men whose names are not only well known in America, but in some cases whose good report has also reached us in this country. These articles are edited by Drs. Peterson and Haines, and the work is produced for the purpose of giving to the medical profession in America a fairly wide view of the subject.

Each chapter is fairly well brought up to date, and in some cases a good deal of fresh information is added. Dr. Ludvig Hektoen has a good chapter on *Post-Mortem* Examinations, which is useful, and in the form given is unusual in works of this sort. Dr. James Ewing gives, among other matter, a good *résumé* of Dr. Galton's identification by the patterns of the papillæ on the finger tips, as well as of Bertillon's system of bone measurements for the same purpose. We are surprised to find in Dr. Allen J. Smith's paper on Asphyxia no mention of death from acetylene gas, several cases having occurred from this most dangerous vapour, and its extended use makes it a matter of importance. The medico-legal aspect of Vision and "Audition" are treated by Dr. E. Jackson; and Speech Disorders by Dr. F. W. Langdon, whilst the "Stigmata of Degeneration" is usefully treated by Dr. Frederick Peterson. These last three subjects have not yet found a large place in our English text-books. Insanity, Idiocy, and Sexual Perversion occupy three chapters, the last making much too much of Dr. Krafft-Ebing's doubtful theories. There is a chapter on Inebriety, in which the subject is treated more fully than usual.

We do not think this work will displace our standard authors on the subject, but it may be found a useful book of reference in the library, especially on the newer subjects.

